# Upper Gastrointestinal Bleeding: A Potential Precursor to Bouveret’s Syndrome

**DOI:** 10.7759/cureus.14368

**Published:** 2021-04-08

**Authors:** Daniela Goyes, Hirsh D Trivedi

**Affiliations:** 1 Internal Medicine, Loyola Medicine MacNeal Hospital, Berwyn, USA; 2 Division of Gastroenterology and Hepatology, Beth Israel Deaconess Medical Center, Harvard Medical School, Boston, USA

**Keywords:** bouveret’s syndrome, upper gastrointestinal bleeding

## Abstract

Bouveret’s syndrome is a rare complication of cholelithiasis. It is characterized by a gallstone entering the intestine through a cholecystoenteric fistula, impacting the duodenum and causing gastric outlet obstruction. Rarely, it presents with hematemesis and melena. The diagnosis involves computed tomography (CT) and the treatment depends on the patient’s stability, the location of the obstruction, stone size, and the fistula. Endoscopy or minimally invasive lithotripsy can be considered initially. If this fails, surgical intervention is recommended. We present a case of upper gastrointestinal bleeding (UGIB) preceding the development of Bouveret’s syndrome.

## Introduction

Gallstone ileus is a complication of cholelithiasis and an uncommon cause of bowel obstruction [1]. Gallstones may enter the intestine through a cholecystoenteric fistula that subsequently impacts the intestinal tract [1-2]. When the stone becomes lodged in the duodenum, it is known as Bouveret's syndrome [3], a rare form of gallstone ileus. Its frequency ranges from 1% to 4%, and its clinical presentation is usually nonspecific [2]. Symptoms such as nausea, vomiting, and abdominal pain have been reported [2,4]. We present a case of upper gastrointestinal bleeding (UGIB) as a presentation of gallstone ileus preceded by the development of a cholecystoenteric fistula.

## Case presentation

An 88-year-old female with a history of hypertension, Alzheimer's disease, and hypothyroidism presented with coffee-ground emesis and melena. Examination revealed tachycardia with a heart rate of 120 beats per minute and hypotension with a blood pressure of 80/50 mmHg. The abdomen was soft, non-distended, and had normoactive bowel sounds. She was intubated given concern for UGIB and lack of airway protection.

**Figure 1:**
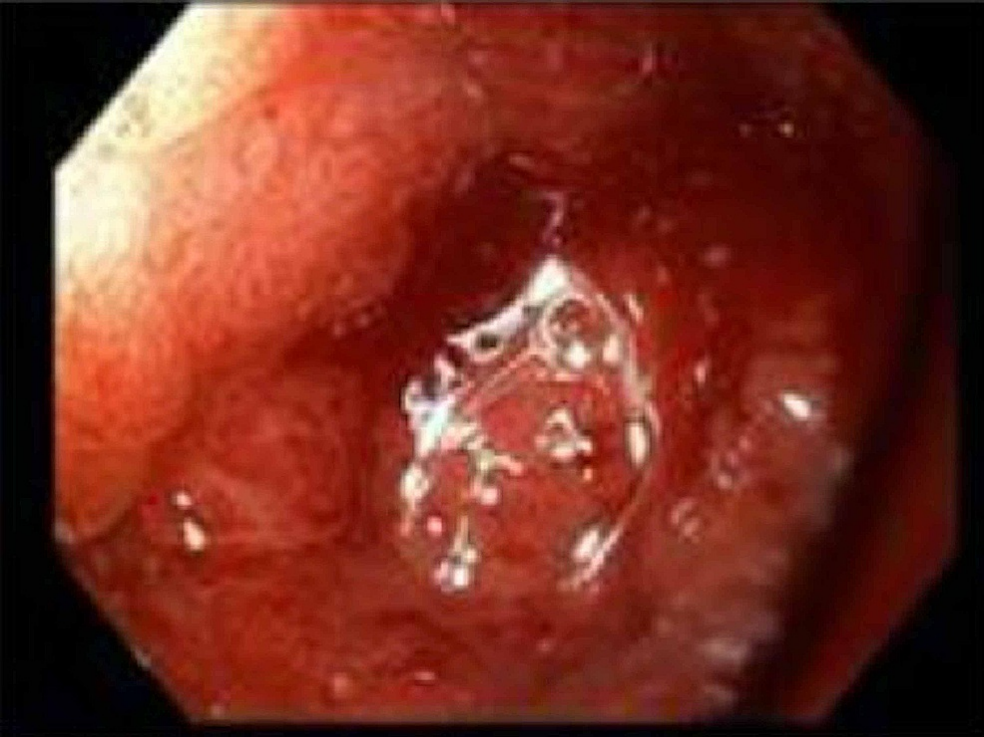
Esophagogastroduodenoscopy showing stenosis and friability at the duodenal bulb

**Figure 2:**
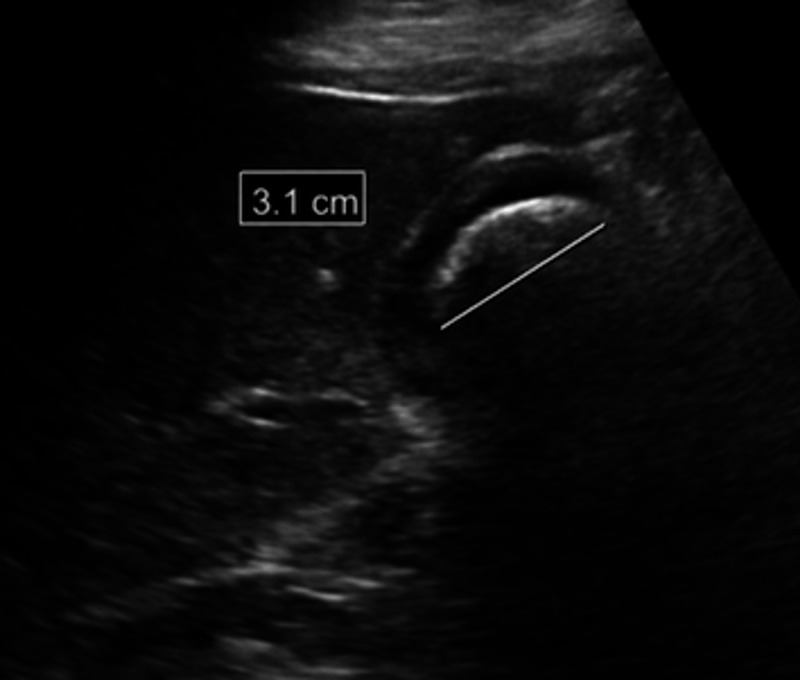
Gallbladder ultrasound showing a large gallstone (3.1 cm) filling the lumen of the gallbladder

**Figure 3:**
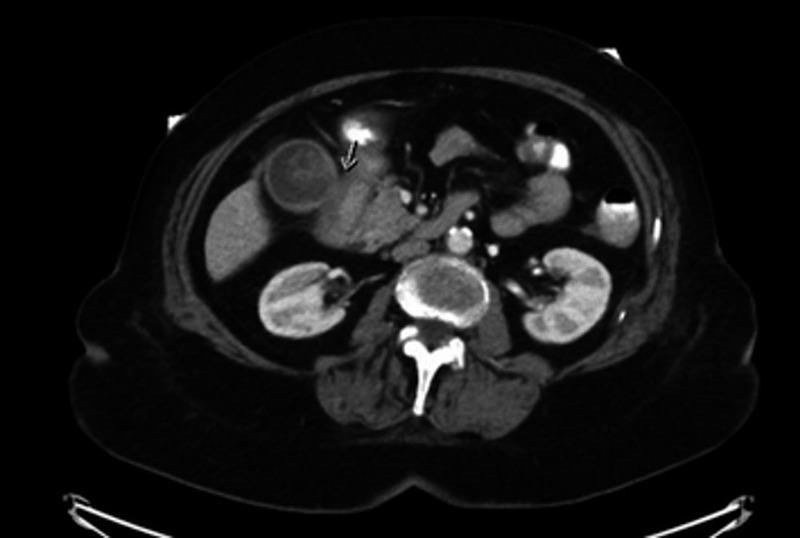
Abdominal computed tomography showing inflammatory changes with mural thickening of the duodenal wall

**Figure 4:**
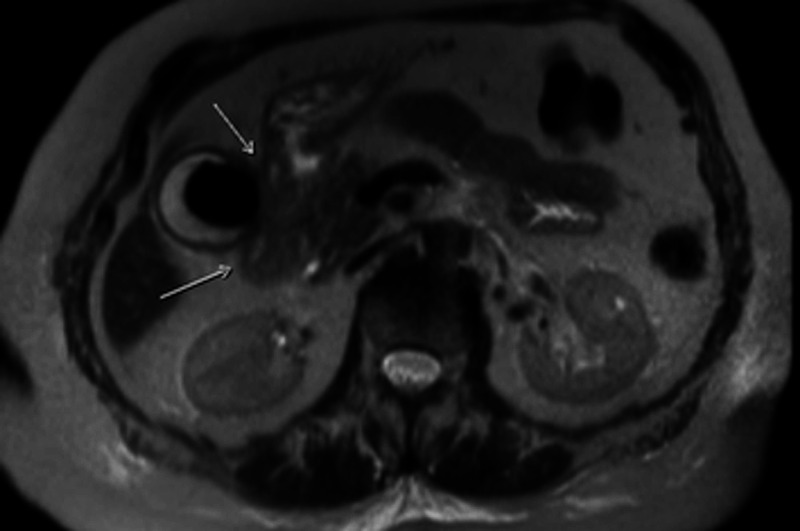
Magnetic resonance cholangiopancreatography showing ongoing gallstone erosion through the gallbladder wall

Laboratory studies showed a white blood cell (WBC) count of 18.9 K/uL, hemoglobin 9.6 g/dL, platelet count of 429 K/uL, international normalized ratio (INR) of 1.3, creatinine 1.3 mg/dL, and lactate 2.9 mmol/L. Esophagogastroduodenoscopy (EGD) demonstrated inflammation, erosion, and friability of the duodenal bulb (Figure 1). The start of the sweep was stenosed secondary to inflammation and it heaped up mucosa. However, the scope was able to pass with gentle manipulation. A large gallstone measuring 3.1 cm filling the lumen of the gallbladder was visualized on the gallbladder ultrasound (Figure 2). Abdominal computed tomography (CT) with contrast (Figure 3) and magnetic resonance cholangiopancreatography (MRCP) (Figure 4) demonstrated pericholecystic inflammatory changes involving the duodenal wall with marked mural thickening of the second part of the duodenum, findings that were concerning for gallstone erosion through the gallbladder wall. However, no definite cholecystoduodenal fistula was demonstrated.

The patient underwent surgical management with laparoscopy, which was converted to open cholecystectomy upon discovery of adherent gallbladder to the adjacent eroded duodenum. Ultimately, a subtotal cholecystectomy was performed, as the strong inflammatory apposition of the tissues posed an increased risk of duodenal injury. A nasogastric tube and surgical drain were temporarily placed and removed upon the improvement in the patient’s symptoms. One week later, the patient had no further evidence of bleeding and was discharged from the hospital.

## Discussion

The prevalence of Bouveret’s syndrome is highest among elderly women (>70 years) with a female to male ratio of 1.9 [5]. It is associated with large gallstones (2-8 cm) and recurrent episodes of acute cholecystitis [5-6]. Large gallbladder stones increase intraluminal pressure, which results in ischemia of the gallbladder wall, allowing erosion through the wall and passing into the adjacent viscera [7]. The clinical presentation is variable. However, there have been rare reports of UGIB [8]. A review of 128 cases identified that the most common signs and symptoms were nausea, vomiting, abdominal pain, anorexia, and gastric or duodenal obstruction. On the other hand, only 15% of patients presented with hematemesis and 6% with melena, clinical manifestations suggestive of UGIB [4,9].

Given the concern for UGIB in our patient, an EGD was performed. The bleeding source was suspected to be secondary to the erosive changes in the duodenum, as alternative causes, such as gastric or duodenal ulcers, were ruled out. If the inflammatory changes involving the duodenal wall were allowed to persist, there would likely have been an eventual development of a cholecystoduodenal fistula leading to Bouveret’s syndrome. Hence, UGIB can rarely present as a manifestation of gallstone ileus preceding the development of Bouveret’s syndrome.

Ultrasound can be useful to show biliary pathology [10]. However, CT is the modality of choice, with a sensitivity of 93% and specificity of 100% [6]. When evaluating for Bouveret’s syndrome, the presence of a fistula can be confirmed by leakage of contrast from the biliary system into the gastrointestinal tract using a CT scan [1]. CT is useful to look for Rigler’s triad that is specific to gallstone ileus, which includes small bowel obstruction, pneumobilia, and ectopic gallstone [5]. The treatment depends on the patient’s initial presentation, location of the obstruction, size of the stone, and presence of a fistula. EGD or minimally invasive lithotripsy should be considered initially. Characteristics such as stomach dilation, duodenal ulcer, cholecystoduodenal fistula, and hard, non-fleshy mass at the obstruction could be visualized on the EGD and are helpful in the diagnosis of Bouveret’s syndrome [5]. However, only 10% of stones can be removed endoscopically [2]. As in our case, 31% of cases can occur with obstruction without evidence of stone or fistula because either the gallstone is compressing the lumen or they are only partially visualized through the wall [5]. If EGD fails, surgical intervention should be performed [11]. Treatment with enterotomy and stone extraction carries a lower mortality rate when compared to segmental bowel resection and fistula closure [12]. The majority of experts recommend exploratory laparotomy and enterolithotomy/bowel resection alone for sicker patients. However, the concerns of enterolithotomy alone are recurrent gallstone ileus and cholangitis [2]. On the other hand, exploratory laparotomy and enterolithotomy/bowel resection, cholecystectomy, and closure of cholecystoenteric fistula (one-stage procedure) are reserved for hemodynamically stable patients [2].

## Conclusions

Our case highlights the importance of recognizing UGIB as a precursor of gallstone. This recognition remains essential in the elderly population in whom the presence of comorbidities places them at a higher risk of surgical complications.
